# Discovery of carbon-vacancy ordering in Nb_4_AlC_3–*x*_ under the guidance of first-principles calculations

**DOI:** 10.1038/srep14192

**Published:** 2015-09-21

**Authors:** Hui Zhang, Tao Hu, Xiaohui Wang, Zhaojin Li, Minmin Hu, Erdong Wu, Yanchun Zhou

**Affiliations:** 1 Shenyang National Laboratory for Materials Science, Institute of Metal Research, Chinese Academy of Sciences, 72 Wenhua Road, Shenyang 110016, China; 2 University of Chinese Academy of Sciences, Beijing 100049, China; 3 Science and Technology on Advanced Functional Composite Laboratory, Aerospace Research Institute of Materials & Processing Technology, No.1 South Dahongmen Road, Beijing 100076, China

## Abstract

The conventional wisdom to tailor the properties of binary transition metal carbides by order-disorder phase transformation has been inapplicable for the machinable ternary carbides (MTCs) due to the absence of ordered phase in bulk sample. Here, the presence of an ordered phase with structural carbon vacancies in Nb_4_AlC_3–*x*_ (*x* ≈ 0.3) ternary carbide is predicted by first-principles calculations, and experimentally identified for the first time by transmission electron microscopy and micro-Raman spectroscopy. Consistent with the first-principles prediction, the ordered phase, o-Nb_4_AlC_3_, crystalizes in *P*6_3_/*mcm* with *a* = 5.423 Å, *c* = 24.146 Å. Coexistence of ordered (o-Nb_4_AlC_3_) and disordered (Nb_4_AlC_3–*x*_) phase brings about abundant domains with irregular shape in the bulk sample. Both heating and electron irradiation can induce the transformation from o-Nb_4_AlC_3_ to Nb_4_AlC_3–*x*_. Our findings may offer substantial insights into the roles of carbon vacancies in the structure stability and order-disorder phase transformation in MTCs.

Binary transition metal carbides have an extraordinary ability to accommodate metalloid structural vacancies (different from the Frenkel, Schottky and anti-Schottky defects, structural vacancies change the stoichiometry of the crystal^1^) in either a disordered or ordered manner. Ordering of carbon vacancies in the binary carbides (NbC_1–*x*_, VC_1–*x*_, TaC_1–*x*_, *etc*.) could give rise to self-organized nanolamellar phases or domains, offering huge opportunities to modulate the microstructure-property space^1^,^2^,^3^,^4^,^5^.

Machinable ternary carbides (MTCs, having a general chemical formula M_*m*_A_*n*_C_*m*–*n*_, where M is an early transition metal element; A is an A group element; *m* and *n* are integers, *m* ≥ 2*n*)^6^,^7^,^8^,^9^,^10^ are the most investigated carbides in the last two decades. Crystallizing in the *P*6_3_/*mmc* (for *n* = 1)^6^ or *R*3–*m* (for *n* = 2)^10^,^11^ space group, their crystal structures are closely related, which can be regarded as the periodically stacking of strongly bonded “M_*m*_C_*m*–*n*_” sheets and “A” atomic layers along [0001]. The building block of “M_*m*_C_*m*–*n*_” in the MTCs strongly resembles that in the binary carbides. The presence of structural carbon vacancies in the MTCs are widely postulated^12^ since monolithic MTCs can be synthesized only with certain degree of carbon deficiency^13^. The carbon vacancies have been believed to be disordered before the pioneering work by Etzkorn and coworkers^14^ on V_4_AlC_3–*x*_ single crystal (with a dimension of 0.2 × 0.2 × 0.01 mm) grown by the auxiliary metal bath technique. They pointed out that the V_4_AlC_3–*x*_ single crystal grown at 1500 °C holds 10% disordered carbon vacancies, while the carbon vacancies become ordered at 1300 °C, forming V_12_Al_3_C_8_. So far, the knowledge of the carbon vacancies in the interesting MTCs is quite limited^12^.

First-principles calculation is a powerful tool to investigate the point defects, crystal structure and properties of the MTCs^7^,^15^,^16^,^17^,^18^,^19^. However, it is frustrating for the phase stability of stoichiometric Nb_4_AlC_3_. Theoretically, Wang *et al*.^20^ argued that stoichiometric Nb_4_AlC_3_ is unstable and decomposes to Nb_2_AlC and NbC above 57 K. Experimentally, Hu *et al*.^21^ demonstrated that Nb_4_AlC_3_ has a good stability at 2000 K. Since Nb_4_AlC_3_ bears striking resemblance to V_4_AlC_3_, this puzzling and unsolved inconsistence necessitates the revisiting of the crystal structure of Nb_4_AlC_3_ with considerations of carbon vacancies.

Here, under the guidance of the first-principles prediction, an ordered phase bearing structural carbon vacancies in Nb_4_AlC_3–*x*_ (*x* ≈ 0.3), o-Nb_4_AlC_3_ (Nb_12_Al_3_C_8_), is unambiguously identified in experiment, demonstrating the validity to investigate the carbon vacancies in the MTCs with the combination of first-principles calculations, electron diffractometry and Raman spectroscopy.

## Results

### Predication of ordered phase

**Figure 1:**
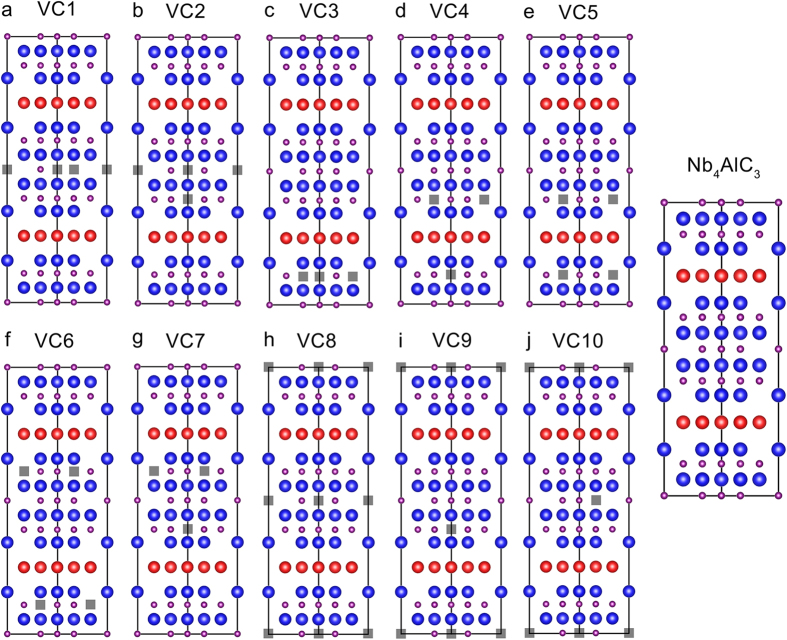
Illustration of constructed VCs. The VCs were constructed by removing two carbon atoms in a 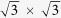 supercell of Nb_4_AlC_3_. (**a**) VC1, (**b**) VC2, (**c**) VC3, (**d**) VC4, (**e**) VC5, (**f**) VC6, (**g**) VC7, (**h**) VC8, (**i**) VC9, (**j**) VC10. As a reference, the projection of Nb_4_AlC_3_ supercell along [112– 0] is provided. The blue, red and purple balls denote the Nb, Al and C atoms. The grey squares denote the carbon vacancies.

In analogy with the carbon-vacancy ordered phase in V_4_AlC_3–*x*_ (Ref. 14)ten hypothetical carbon-vacancy configurations (VCs) with a vacancy concentration of 1/9 were constructed based on a 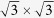 supercell of Nb_4_AlC_3_ (Fig. 1a–j). There are two distinct types of Nb_6_C octahedrons in Nb_4_AlC_3_ (*P*6_3_/*mmc*), involving the carbon atoms located at 4*f* sites with a Nb–C bond length of 2.21 Å (OCT-4*f*) and 2*a* sites with a Nb–C bond length of 2.28 Å (OCT-2*a*). VC1, VC8 and VC10 have two OCT-2*a* type carbon-vacant octahedrons in each unit cell. In contrast, VC3, VC4, VC5, VC6 and VC7 have two OCT-4*f* type carbon-vacant octahedrons. VC2 and VC9 own one OCT-2*a* and one OCT-4*f* type carbon-vacant octahedrons. The crystal structure information of the constructed VCs is provided in Supplementary Table 1. To evaluate the phase stability, the formation energy for VC (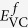) is calculated by 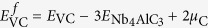. *E*_VC_ and 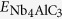 are total energies of the VC and Nb_4_AlC_3_ unit cell, respectively. The chemical potential of carbon, *μ*_C_, is assumed to be that in graphite. Table 1 lists the calculated values. With lower total energies, VC8 and VC10 are the energetically most possible VCs. The 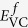 for VC8 and VC10 are negative (a brief discussion in the context of chemical potential is provided in Supplementary Note 1), indicating that stoichiometric Nb_4_AlC_3_ is metastable and prone to spontaneously forming ordered phases. With a more negative 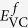, VC8 isostructural with V_12_Al_3_C_8_ (Ref. 14) is slightly more favorable than VC10 from an energetic point of view. The mechanical stability of crystals requires the elastic constants *c*_*ij*_ to match the Born–Huang criterion. Specifically, the restrictions for hexagonal crystal system^22^,^23^ are: 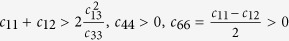. According to the calculated elastic constants in Supplementary Table 2, VC8 and VC10 satisfy the mechanical stability criteria. In addition, the phonon dispersion curves in Supplementary Fig. 1 have no imaginary frequencies. VC8 and VC10 are therefore dynamically stable.

### **I**dentification of ordered phase and determination of crystal structure

**Figure 2:**
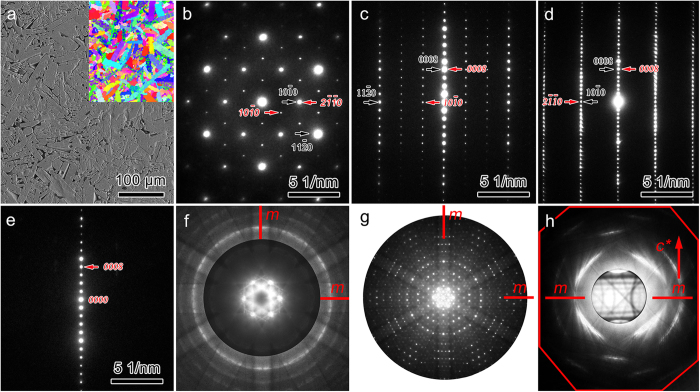
Grain morphology and EDPs. (**a**) Scanning electron microscopy image of the as-prepared Nb_4_AlC_3–*x*_. Inset is an electron backscatter diffraction image. The grains are elongated. Selected area EDPs belonging to (**b**) [0001], (**c**) [12– 10], (**d**) [01– 10] and (**e**) [*hki*0] of o-Nb_4_AlC_3_ are indexed in red fonts. The diffraction spots marked by black arrows coincide with those of Nb_4_AlC_3–*x*_, as denoted with black indices. CBED patterns were collected along (**f**,**g**) [0001] and (**h**) [*hki*0]. (**f**) and (**g**) were recorded with different convergence angles. A (0000) CBED disk was montaged in (**h**). “*m*” and “c*” stand for mirror plane and [0001] direction in reciprocal space, respectively.

**Figure 3:**
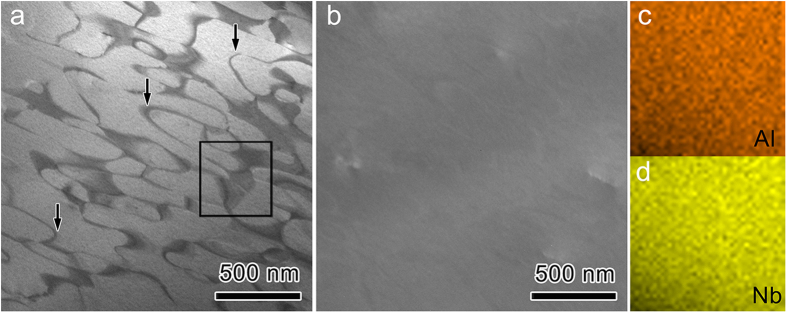
Coexistence of o-Nb_4_AlC_3_ and Nb_4_AlC_3–*x*_ and composition analysis. TEM dark field images were recorded with (**a**) (101– 0) and (**b**) (303– 0) of o-Nb_4_AlC_3_. EDS mapping in the squared region in (**a**) with (**c**) Al and (**d**) Nb.

To test the theoretical prediction, the phase component of the as-prepared Nb_4_AlC_3–*x*_ (*x* ≈ 0.3) was revisited. Like other MTCs, grains of Nb_4_AlC_3–*x*_ are elongated with *ca*. 54 μm in length and 10 μm in width, as shown in Fig. 2a. Indexing the low-index zone axis electron diffraction patterns (EDPs) in Fig. 2b–d results in a new hexagonal structure with *a* = 5.5 Å, *c* = 25.2 Å, which is different from that of previously reported Nb_4_AlC_3_ (*a* = 3.1 Å, *c* = 24.1 Å)^24^. For convenience, the new phase is denoted as o-Nb_4_AlC_3_. Then, Fig. 2b–d correspond to the EDP of [0001], [12– 10] and [01– 10] of o-Nb_4_AlC_3_, respectively. The reflection conditions are *l* = 2*n* for (*h*h– 0*l*) and (000*l*). The appearance of (000*l*) with *l* = 2*n* *+* 1 in Fig. 2d is caused by double diffractions, which can be verified by its disappearance in the EDP collected along [*hki*0] (Fig. 2e). The convergent beam electron diffraction (CBED) patterns with different convergence angles along [0001] in Fig. 2f,g demonstrate that there is a six-fold rotation axis and two mirror planes along [0001]. In addition, the CBED pattern collected along [*hki*0] in Fig. 2h indicates a mirror plane vertical to [0001]. Therefore, the corresponding point group is 6/*mmm*. Considering the reflection conditions, the space group of o-Nb_4_AlC_3_ is determined to be *P*6_3_/*mcm*. Noteworthily, the diffraction spots marked by black arrows in Fig. 2b–d can be indexed with Nb_4_AlC_3–*x*_ as well. Figure 3a,b present transmission electron microscopy (TEM) dark field morphologies imaged with (101–0) and (303– 0) of o-Nb_4_AlC_3_, respectively. Since (303– 0) of o-Nb_4_AlC_3_ coincides with (112– 0) of Nb_4_AlC_3–*x*_ (Fig. 2b,c), the dark domains with irregular shape in Fig. 3a are Nb_4_AlC_3–*x*_; while the regions with bright contrasts correspond to o-Nb_4_AlC_3_. The worm-like ribbons marked by arrows are antiphase boundaries in o-Nb_4_AlC_3_.

**Figure 4:**
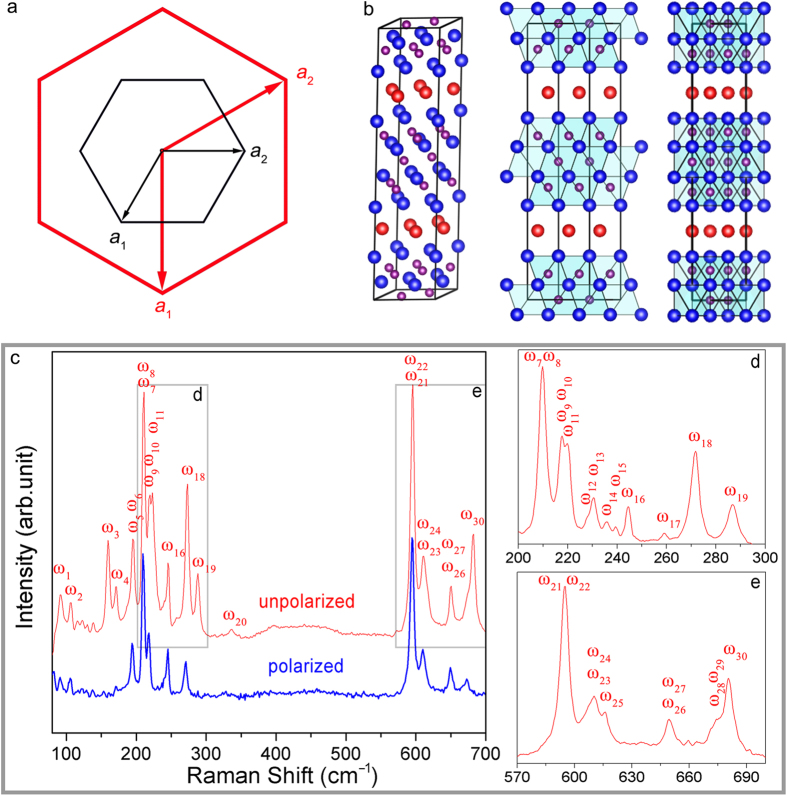
Crystal structure and Raman spectrum of o-Nb_4_AlC_3_. (**a**) Illustration of the unit-cell projection of o-Nb_4_AlC_3_ (red) and Nb_4_AlC_3–*x*_ (black) along [0001]. The EDPs in Fig. 2c,d demonstrate that the lengths of ***c*** axis of o-Nb_4_AlC_3_ and Nb_4_AlC_3–*x*_ in the present experiment are identical. (**b**) Unit cell of o-Nb_4_AlC_3_ (left) and the projection of edge-sharing Nb_6_C octahedrons along [01– 10] (median) and [12– 10] (right). The octahedral interstitial sites at the origin and (0, 0, 1/2) are not occupied by carbon atoms. The blue, red and purple balls illustrate the Nb, Al and C atoms. (**c**) Polarized and unpolarized Raman spectrums collected with a 600 lines per mm diffraction grating. (**d**,**e**) Unpolarized Raman spectrums collected with a 1800 lines per mm diffraction grating in the wavenumber ranges boxed in (**c**).

As determined by electron-probe X-ray microanalysis, the molar ratio of Nb:Al in o-Nb_4_AlC_3_ is 4:1.05 (see Supplementary Table 3). The energy dispersive X-ray spectroscopy (EDS) mapping of Al (Fig. 3c) and Nb (Fig. 3d) demonstrates that there is no compositional difference of Al and Nb between o-Nb_4_AlC_3_ and Nb_4_AlC_3–*x*_. In addition, carbon is 10% deficient in the starting materials to synthesize monolithic Nb_4_AlC_3_^24^. Then, the chemical formula of o-Nb_4_AlC_3_ is Nb_24_Al_6+δ_C_18–*n*_ (δ = 0.3) since the unit cell of o-Nb_4_AlC_3_ is three times that of Nb_4_AlC_3–*x*_ (Fig. 4a). As the lowest multiplicity for the Wyckoff sites of *P*6_3_/*mcm* (Ref. 25) is 2, the formation of o-Nb_4_AlC_3_ cannot be caused by an excess of Al, otherwise the minimum Al/Nb is 8/24 with δ = 2. Considering the nominal composition of the starting materials (Nb:C = 12:8.1) and restrictions on the Wyckoff sites of *P*6_3_/*mcm* (Ref. 25), o-Nb_4_AlC_3_ is Nb_12_Al_3_C_8_ with the carbon vacancies occupying the 2*b* Wyckoff site. Namely, o-Nb_4_AlC_3_ has the VC8 configuration. The experimental EDPs and simulated patterns with the VC8 configuration are in excellent consistence (see Supplementary Fig. 2a–f). The crystal structure information of o-Nb_4_AlC_3_ is listed in Table 2. o-Nb_4_AlC_3_ can be constructed readily by removing the carbon atoms at (0, 0, 0) and (0, 0, 1/2) of the 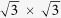 supercell (Fig. 4b). The orientation relationship is: [12– 10] o-Nb_4_AlC_3_ ‖ [11–00] Nb_4_AlC_3–*x*_, [01–10] o-Nb_4_AlC_3_ ‖ [12– 10] Nb_4_AlC_3–*x*_.

### Raman spectroscopic verification of o-Nb_4_AlC_3_

To further verify the crystal structure, micro-Raman spectroscopic investigations were conducted. The polarized and unpolarized Raman spectra are presented in Fig. 4c–e. The group theory predicts the following symmetries for zone-center (Γ point) optical phonons: Γ_optical_ = 7*A*_1g_ + 3*A*_1u_ + 5*A*_2g _+ 7*A*_2u _+ 4*B*_1g_ + 8*B*_1u_ + 7*B*_2g_ + 4*B*_2u_ + 11*E*_2u_ + 12*E*_2g_ + 11*E*_1u_ + 11*E*_1g_, where *A*_1g_, *E*_1g_ and *E*_2g_ are Raman active modes. The experimental and theoretical Raman shifts calculated by first-principles (Table 3) are well consistent. In addition, the peaks located at 158 (ω_3_), 169 (ω_4_), 220 (ω_11_), 259 (ω_17_), 287 (ω_19_), 616 (ω_25_) and 681 cm^–1^ (ω_30_) disappear in the polarized Raman spectrum, indicating a symmetry of *A*_1g_ (Ref. 11), which is exactly the same with that predicted by the first-principles calculations. Therefore, the Raman spectroscopic investigation unambiguously validates the crystal structure of o-Nb_4_AlC_3_ with the predicted VC8 configuration.

**Figure 5:**
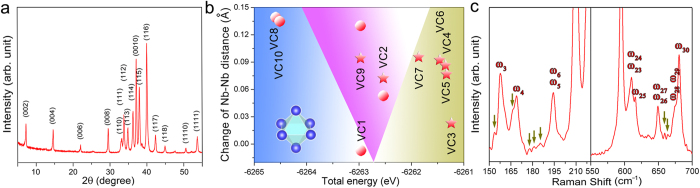
XRD pattern, dilatation of the carbon-vacant octahedron and extra Raman peaks. (**a**) XRD pattern of the as-prepared Nb_4_AlC_3–*x*_. All identifiable peaks belonging to o-Nb_4_AlC_3_ and Nb_4_AlC_3–*x*_ coincide. For the sake of conciseness, they are indexed with the cell of o-Nb_4_AlC_3_. (**b**) Change of diagonal distances of Nb (blue balls in the inset) in the carbon-vacant octahedron using the values of Nb_6_C octahedrons as references. Red balls and stars denote the octahedrons with a carbon vacancy located at the 2*a* and 4*f* sites of Nb_4_AlC_3–*x*_ (*P*6_3_/*mmc*), respectively. (**c**) Raman spectrum collected with a 1800 lines per mm diffraction grating. Arrows denote the extra weak Raman peaks not belonging to o-Nb_4_AlC_3_ or Nb_4_AlC_3–*x*_. Those peaks are believed to be generated by the vibration of VC10.

Statistics on the TEM dark field morphologies imaged with superlattice diffraction spots indicate that o-Nb_4_AlC_3_ accounts for *ca*. 81 vol.% of the as-prepared sample. The X-ray diffraction (XRD) pattern in Fig. 5a is indexed with o-Nb_4_AlC_3_. The superlattice peaks, (*h*0h–l) with *h* = 3*n* ± 1, are unidentifiable in the XRD pattern due to their remarkably low intensities (see Supplementary Table 4).

## Discussion

Formation of a carbon vacancy within the Nb_6_C octahedron breaks six Nb–C bonds and destabilizes the structure. Meanwhile, the redistribution of the electron charge within the vacancy neighbors through the dilatation of the carbon-vacant octahedron strengthens the remaining Nb–C bonds around the carbon vacancy and stabilizes the structure. The triumph of the stabilizing factor over the destabilizing one gives rise to carbon-vacancy ordered phases^26^,^27^. With weaker Nb–C bonds, forming a carbon vacancy in OCT-2*a* costs less energy than that in OCT-4*f* (Fig. 5b). Similar features have been confirmed in Ta_4_AlC_3_ and Ti_4_AlN_3_ (Ref. 16,17). Generally speaking, the more the diagonal distances of the Nb atoms in the carbon-vacant octahedron expand, the stronger the remaining Nb–C bonds around the carbon vacancy become, and then the more stable the carbon-vacant structure is (Fig. 5b). Therefore, VC8 and VC10 with carbon vacancies only in OCT-2*a* and most expansions of the carbon-vacant octahedrons have lower total energies than the other VCs.

The revisiting of the phase component in Nb_4_AlC_3–*x*_ confirms the presence of carbon-vacancy ordered phase predicted by our first-principles calculations: o-Nb_4_AlC_3_ has the VC8 configuration. The difference of 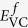 between VC10 and VC8 is only 0.07 eV, and it is therefore not unreasonable to anticipate the existence of VC10. Virtually, there are several weak Raman peaks belonging to neither o-Nb_4_AlC_3_ (VC8) nor Nb_4_AlC_3–*x*_ in the Raman spectrum collected with a 1800 lines per mm diffraction grafting (Fig. 5c). These extra peaks are most likely generated by VC10 (see Supplementary Table 5). Since no EDPs belonging to VC10 (see Supplementary Fig. 2g–i) were identified in the present study, VC10 is believed to exist not in a highly ordered manner. The crystal structure information of VC10 is provided in Supplementary Table 6.

**Figure 6:**
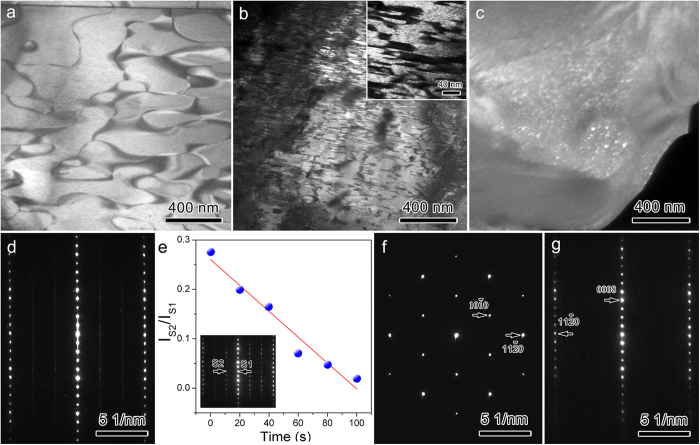
Domain morphologies and electron irradiation. TEM dark field image recorded with (101– 0) of o-Nb_4_AlC_3_ in the sample (**a**) as-prepared, (**b**) quasi-quenched from 1400 °C after dwelling for 30 min, and (**c**) quasi-quenched from 1500 °C after dwelling for 10 s. o-Nb_4_AlC_3_ and Nb_4_AlC_3–*x*_ are in bright and dark contrasts, respectively. Inset in (**b**) is an enlarged morphology demonstrating the disordered nanodomains. (**d**) An EDP with the features of short-range ordering. The superlattice diffraction spots become weak streaks. (**e**) Dependence of I_S2_/I_S1_ on the irradiation time. I_S1_ and I_S2_ are the intensities of the diffraction spots marked in the inset. (**f,g**) EDPs recorded after irradiated 120 s. (**f**,**g**) belong to [0001] and [11– 00] of Nb_4_AlC_3–*x*_, respectively. The electron dose for irradiation is approximately 0.04 e Å^–2^ s^–1^.

Carbon-vacancy ordered phase is stable at low temperature^1^. When temperature rises and the contribution of entropy to the Gibbs free energy is appreciable, carbon vacancies tend to be in short-range order or disordered. Therefore, o-Nb_4_AlC_3_ is a low-temperature phase; while Nb_4_AlC_3–*x*_ (with certain amounts of disordered carbon vacancies) is the corresponding high-temperature phase. As indicated by the first-principles calculations (Table 1, Fig. 5b) and Rietveld refinements of X-ray (neutron) diffraction patterns of V_4_AlC_3–*x*_ (Ti_4_AlN_3–*x*_)^14^,^28^, the disordered vacancies in Nb_4_AlC_3–*x*_ are most likely located at the 2*a* site of the *P*6_3_/*mmc* space group. The existence of carbon-vacancy disordered Nb_4_AlC_3–*x*_ at room temperature is due to the fact that the cooling rate during the sample synthesis is not slow enough, and brings about disordered domains (Figs 3a and 6a). When o-Nb_4_AlC_3_ is heated above a critical temperature, transformation to Nb_4_AlC_3–*x*_ occurs with the nucleation and growth of new disordered nanodomains in the ordered phase (Fig. 6b). For the sample quasi-quenched from 1400 °C after keeping 30 min, the amount of disordered domains (with dark contrasts) increases from *ca*. 19 vol.% (Fig. 6a) to 36 vol.% (Fig. 6b). Dwelling for 10 s at 1500 °C, nearly all o-Nb_4_AlC_3_ transforms to Nb_4_AlC_3–*x*_, leaving some ordered nanodomains (with bright contrasts, Fig. 6c). Consequently, the EDP (Fig. 6d) exhibits the features of short-range ordering. Thereby, similar to the carbon-vacancy disordering in V_4_AlC_3–*x*_ where the disordering occurs in the range from 1300 °C to 1500 °C^14^, that in Nb_4_AlC_3–*x*_ starts around 1400 °C and completes at 1500 °C.

Resembling the ordered phase in binary carbides^29^,^30^, transformation from o-Nb_4_AlC_3_ to Nb_4_AlC_3–*x*_ can be induced by electron irradiation, as shown in Supplementary Movies 1,2. The intensity of the superlattice diffraction spots decreases dramatically as the irradiation proceeds (Fig. 6e). Irradiated for approximately 120 s, the superlattice diffraction spots disappear (Fig. 6f,g) with the transformation from o-Nb_4_AlC_3_ to Nb_4_AlC_3–*x*_. The extremely electron irradiation sensitive nature possibly hides o-Nb_4_AlC_3_ and domains from being discovered before.

In summary, under the guidance of first-principles calculations, a new carbon-vacancy ordered phase, o-Nb_4_AlC_3_ (Nb_12_Al_3_C_8_) has been discovered. It crystalizes in the space group of *P*6_3_/*mcm* with *a* = 5.423 Å, *c* = 24.146 Å. Coexistence of ordered (o-Nb_4_AlC_3_) and disordered (Nb_4_AlC_3–*x*_) phase brings about domains with irregular shape. Both heating and electron irradiation can induce the transformation from o-Nb_4_AlC_3_ to Nb_4_AlC_3–*x*_. The excellent consistency between the first-principles prediction and experimental results demonstrated in this work may inspire the theoretical investigation on the vacancies in over 70 machinable ternary carbides/nitrides. The unveiled domain structure likely ignites investigation enthusiasm on the order-disorder phase transformation as well.

## Methods

### First-principles calculations with CASTEP module

Electronic exchange-correlation energy was treated under the generalized gradient approximation (GGA–PBE)^31^,^32^. Interaction of electrons with ion cores was represented by norm-conserving pseudopotential^33^. The plane-wave cut off energy and Brillouin zone sampling were fixed at 770 eV and 5 × 5 × 2 Monkhorst-Pack-point meshes^34^, respectively. The Broyden-Fletcher-Goldfarb-Shanno minimization method was used for geometry optimization^35^, where the tolerances were selected as the difference in total energy within 1 × 10^−8^ eV per atom, maximum ionic Hellmann-Feynman force within 0.001 eV Å^−1^, maximum ionic displacement within 5 × 10^−4^ Å, and maximum stress within 0.02 GPa. The elastic constants were determined by the method reported by Milman *et al*.^36^. Vibrational frequencies were determined with the finite displacement method^37^.

### Sample preparation

Bulk Nb_4_AlC_3–*x*_ (*x* ≈ 0.3) sample was synthesized by the method reported in Ref. 21,38. Briefly, Nb (−300 mesh), Al (−300 mesh) and graphite (D_90_ = 6.5 μm) powders with a molar ratio of 4 : 1.2 : 2.7 were homogenized with agate balls and absolute alcohol in an agate jar for 12 h, and then dried at 70 °C for 24 h. After that, the blended powders were cold compressed in a graphite mold. Finally, the green compact together with the mold were put into a hot pressing furnace and sintered at 1900 °C for 1 h under a uniaxial pressure of 30 MPa with flowing Ar as protective gas.

### Composition and microstructure characterization

Composition of thirty points in different regions of the as-prepared sample was analyzed by an electron-probe microanalyser (Shimadzu EPMA-1610, Kyoto, Japan). The phase components were investigated by XRD in an X-ray diffractometer (Rigaku D/max-2400, Tokyo, Japan) with Cu K*α* radiation. The microstructural characterizations were performed on a transmission electron microscope (FEI Tecnai G^2^ F20, Oregon, USA) working at 200 kV with an energy dispersive spectroscopy detector and a high-angle annular dark-field detector in the scanning transmission electron microscopy system. Selected area electron diffraction and CBED patterns were taken in Tecnai T12 (FEI Tecnai T12, Oregon, USA).

### Micro-Raman spectroscopic characterization

Unpolarized and polarized Raman spectrums were collected at room temperature on a LabRAM HR800 (Horiba Jobin Yvon, France) equipped with an air-cooled CCD array detector in a backscattering geometry, and with diffraction gratings of 600 and 1800 lines per mm. A He–Ne laser (632.82 nm) with an incident power of *ca*. 20 mW was used as excitation source. Theoretical Raman shifts were obtained by lattice dynamics calculation. According to Zhang *et al*.^11^, the peaks with *A*_1g_ symmetry disappear when 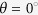 (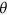 is the angle between the ***z*** axis of hexagonal crystal and that of the system coordinates). Therefore, the grains with 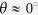 were chosen to collect the polarized and unpolarized Raman spectrums.

### Quasi-quenching

Quasi-quenching was realized by a thermomechanical simulator, Gleeble 3800. The cooling rate above 800 °C is near 200 °C s^–1^, as shown in Supplementary Fig. 3.

## Additional Information

**How to cite this article**: Zhang, H. *et al*. Discovery of carbon-vacancy ordering in Nb_4_AlC_3-x_ under the guidance of first-principles calculations. *Sci. Rep*. **5**, 14192; doi: 10.1038/srep14192 (2015).

## Supplementary Material

Supplementary Information

Supplementary Video S1

Supplementary Video S2

## Figures and Tables

**Table 1:** Total energy and formation energy for various VCs.

Configuration	Total energy (eV)	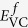 * (eV)
VC1	–6262.961	0.671
VC2	–6262.534	1.098
VC3	–6261.248	2.384
VC4	–6261.351	2.019
VC5	–6261.332	2.280
VC6	–6261.464	2.168
VC7	–6261.864	1.768
VC8	–6264.590	–0.958
VC9	–6262.974	0.658
VC10	–6264.516	–0.884

^*^Formation energy for VC. The total energy of Nb_4_AlC_3_ unit cell and chemical potential of carbon in graphite is –2191.204 and –154.990 eV, respectively.

**Table 2:** Crystal structure information of o-Nb_4_AlC_3_.

	Formula	Nb_12_Al_3_C_8_
	Space group	*P*6_3_*/mcm* (193)
	Methods	First-principles calculation
	Lattice parameters* (Å)	a = 5.489, c = 24.014
**Atom positions**	Nb_1_ (4*e*)	(0, 0, 0.157)
	Nb_2_ (8*h*)	(0.333, 0.667, 0.161)
	Nb_3_ (12*k*)	(0.351, 0, 0.056)
	Al (6*g*)	(0.323, 0, 0.250)
	C_1_ (4*d*)	(0.333, 0.667, 0)
	C_2_ (12*k*)	(0.664, 0, 0.110)

^*^The values determined by electron diffraction and XRD are *a* = 5.5 Å, *c* = 25.2 Å and *a* = 5.423 Å, *c* = 24.146 Å, respectively.

**Table 3:** Experimental and theoretical Raman shifts of o-Nb_4_AlC_3_.

Label	Raman Shift (cm^–1^)	Symmetry	Label	Raman Shift (cm^–1^)	Symmetry
*ω*_1_	90 (73)	(*E*_2g_)	*ω*_16_	245 (264)	(*E*_1g_)
*ω*_2_	106 (104)	(*E*_1g_)	*ω*_17_	259* (265)	*A*_1g_ (*A*_1g_)
*ω*_3_	158 (153)	*A*_1g_ (*A*_1g_)	*ω*_18_	272 (268)	(*E*_2g_)
*ω*_4_	169 (159)	*A*_1g_ (*A*_1g_)	*ω*_19_	287 (279)	*A*_1g_ (*A*_1g_)
*ω*_5_	194 (170)	(*E*_2g_)	*ω*_20_	335 (335)	(*E*_1g_)
*ω*_6_	194 (185)	(*E*_1g_)	*ω*_21_	595 (567)	(*E*_1g_)
*ω*_7_	210 (189)	(*E*_2g_)	*ω*_22_	595 (568)	(*E*_2g_)
*ω*_8_	210 (193)	(*E*_2g_)	*ω*_23_	610 (583)	(*E*_1g_)
*ω*_9_	218 (204)	(*E*_1g_)	*ω*_24_	610 (585)	(*E*_2g_)
*ω*_10_	218 (207)	(*E*_2g_)	*ω*_25_	616* (602)	*A*_1g_ (*A*_1g_)
*ω*_11_	220 (212)	*A*_1g_ (*A*_1g_)	*ω*_26_	650 (636)	(*E*_1g_)
*ω*_12_	228* (223)	(*E*_1g_)	*ω*_27_	650 (637)	(*E*_2g_)
*ω*_13_	230* (229)	(*E*_1g_)	*ω*_28_	674* (680)	(*E*_2g_)
*ω*_14_	236* (230)	(*E*_2g_)	*ω*_29_	674* (681)	(*E*_1g_)
*ω*_15_	240* (238)	(*E*_2g_)	*ω*_30_	681 (686)	*A*_1g_ (*A*_1g_)

The theoretical Raman shifts and symmetry information are provided in the parentheses.

^*^Recorded with a diffraction grating of 1800 lines per mm.
