# Supplementary material for: Discovery of carbon-vacancy ordering in Nb4AlC3–x under the guidance of first-principles calculations
Source: Sci Rep. 2015 Sep 21;5:14192. doi: 10.1038/srep14192 (PMC4585700; doi:10.1038/srep14192)
Supplement: Supplementary Information [file srep14192-s1.doc]

**Supplementary Information**

**Discovery of carbon-vacancy ordering in Nb_4_AlC_3_ under the guidance of first-principles calculations**

Hui Zhang^1,2^, Tao Hu^1,2^, Xiaohui Wang^1^, Zhaojin Li^1,2^, Minmin Hu^1,2^, Erdong Wu^1^ & Yanchun Zhou^3^

^1^Shenyang National Laboratory for Materials Science, Institute of Metal Research, Chinese Academy of Sciences, 72 Wenhua Road, Shenyang 110016, China.

^2^University of Chinese Academy of Sciences, Beijing 100049, China.

^3^Science and Technology on Advanced Functional Composite Laboratory, Aerospace Research Institute of Materials & Processing Technology, No.1 South Dahongmen Road, Beijing 100076, China.

Correspondence and requests for materials should be addressed to Xiaohui Wang (email: wang@imr.ac.cn).

**Supplementary Note 1**

Accurately, the formation energy for the carbon-vacancy ordered configurations (VCs), $E_{\text{VC}}^{f}$, is calculated by $E_{\text{VC}}^{f}=E_{\text{VC}}-3E_{\text{Nb}_{4}\text{AlC}_{3}}+ 2\mu_{\text{C}}\text{+2}\Delta\mu$, $E_{\text{VC}}$and$E_{\text{Nb}_{4}\text{AlC}_{3}}$ are total energies of the VCs and Nb_4_AlC_3_ unit cell, respectively. $\mu_{\text{C}}$ is the chemical potential of C in graphite. $\Delta\mu$ is the difference between the chemical potential of C in actual chemical environment and that in graphite. Since graphite does not precipitate in the actual chemical environment, $\Delta\mu<0$. Therefore, the real values of $E_{\text{VC}}^{f}$are lower than those calculated by assuming the chemical potential of C to be that in graphite. The $E_{\text{VC}}^{f}$for VC8 and VC10 are thereby more negative than those in the paper.

**Supplementary Figures**

**
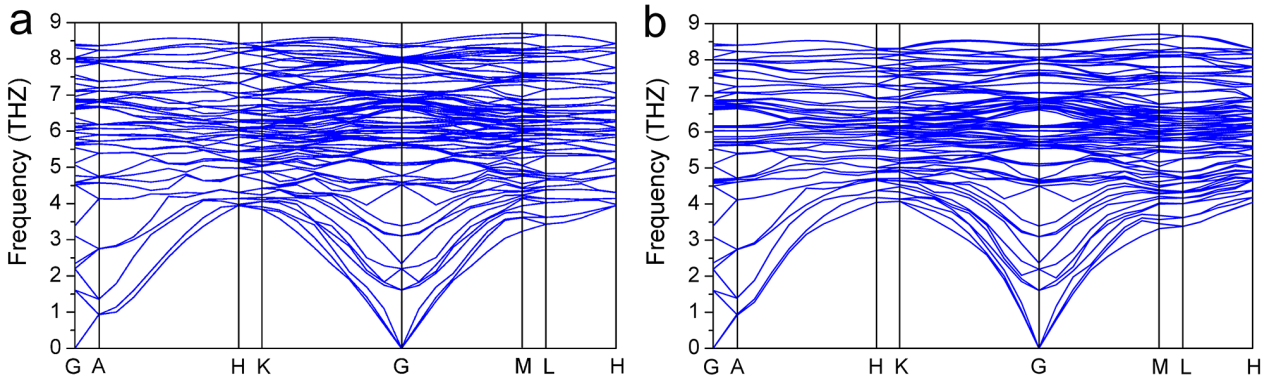
**

**Supplementary Figure 1 |** Phonon dispersion of carbon-vacancy configuration (a) VC8 and (b) VC10.


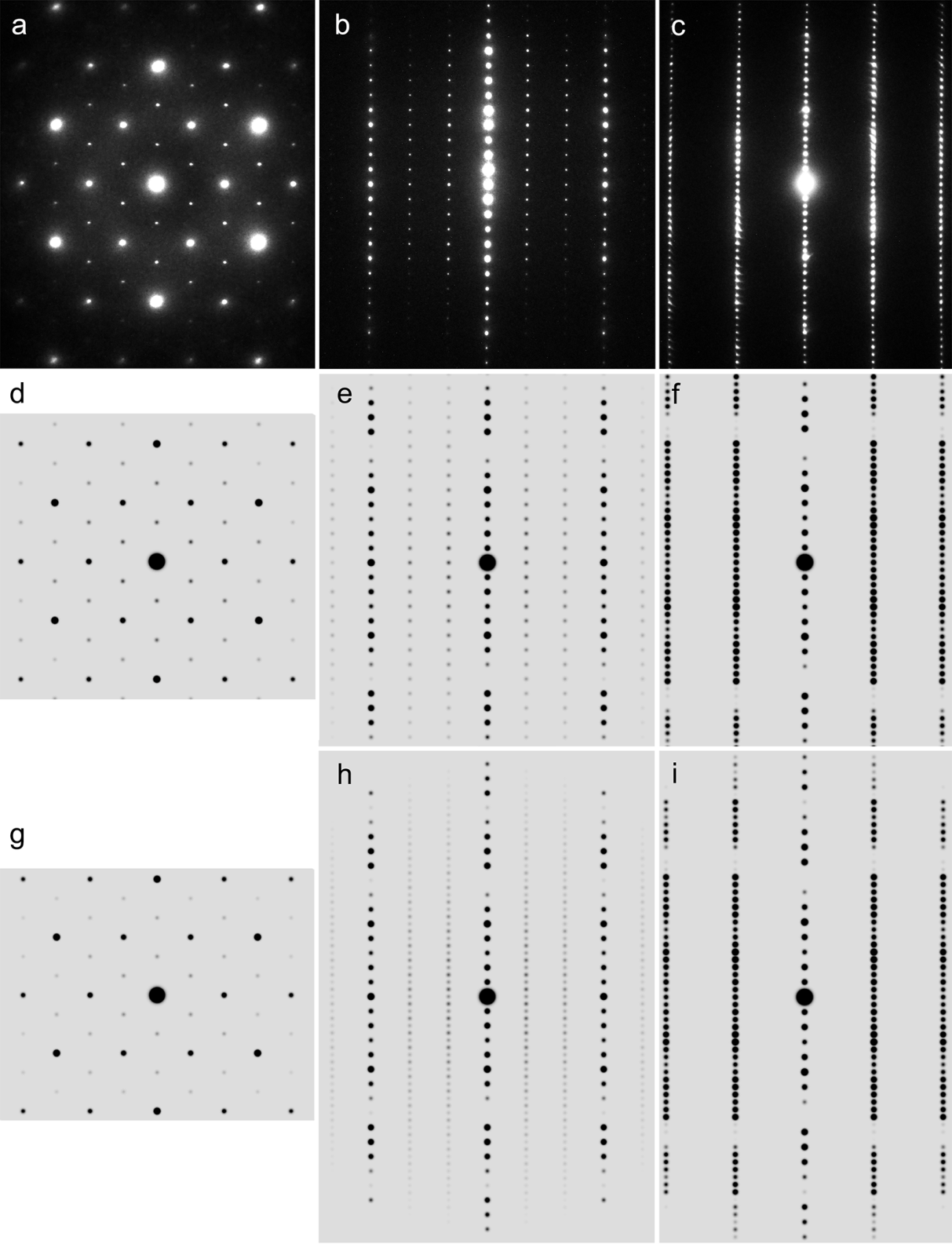


**Supplementary Figure** **2 |** Experimental and simulated electron diffraction patterns (EDPs). (a–c) Experimental EDPs. (d–f) Simulated EDPs of carbon-vacancy configuration VC8 (o-Nb_4_AlC_3_). (g–i) Simulated EDPs of carbon-vacancy configuration VC10.


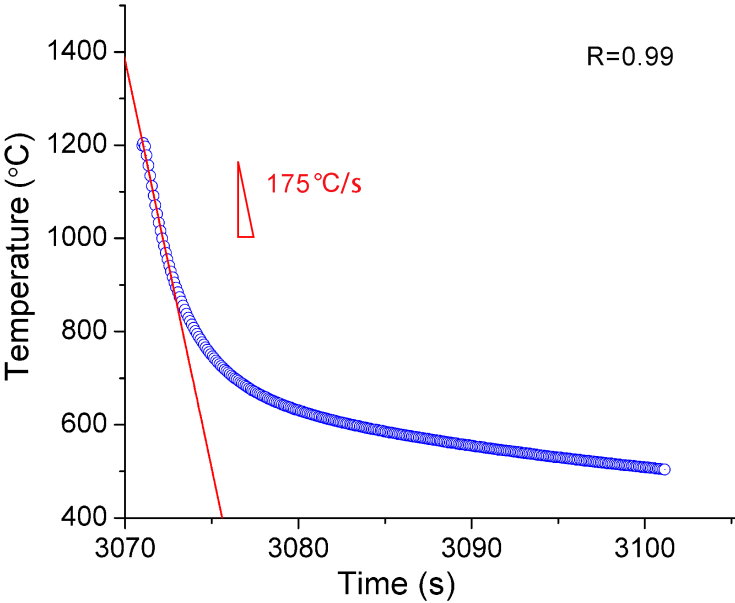


**Supplementary Figure** **3 |** Cooling rate of the quasi-quenching treatment.

**Supplementary Tables**

**Supplementary Table 1 |** Crystal structure information of the constructed$\sqrt{3} \times\sqrt{3}$ supercell of Nb_4_AlC_3_. The C atoms located at C_4_ & C_5_, C_4_ & C_18_, C_7_ & C_9_, C_9_ & C_16_, C_7_ & C_16_, C_7_ & C_10_, C_10_ & C_18_, C_1_ & C_4_, C_1_ & C_18_ and C_1_ & C_5_ are removed to construct carbon-vacancy configurations (VCs) VC1, VC2, VC3, VC4, VC5, VC6, VC7, VC8, VC9 and VC10, respectively.

| **Formula** | | | Nb_24_Al_6_C_18_ | | |
| --- | --- | --- | --- | --- | --- |
| **Space group** | | | *P*1 | | |
| **Lattice parameters (Å)** | | | *a* = 5.4, *b* = 5.4, *c* = 24.1 | | |
| **Atom positions** | Nb_1_ | (0.000, 0.333, 0.055) | | Al_1_ | (0.000, 0.333, 0.250) |
|  | Nb_2_ | (0.333, 1.000, 0.055) | | Al_2_ | (0.333, 1.000, 0.250) |
|  | Nb_3_ | (0.667, 0.667, 0.055) | | Al_3_ | (0.667, 0.667, 0.250) |
|  | Nb_4_ | (0.000, 0.667, 0.555) | | Al_4_ | (0.000, 0.667, 0.750) |
|  | Nb_5_ | (0.667, 1.000, 0.555) | | Al_5_ | (0.667, 1.000, 0.750) |
|  | Nb_6_ | (0.333, 0.333, 0.555) | | Al_6_ | (0.333, 0.333, 0.750) |
|  | Nb_7_ | (0.000, 0.667, 0.945) | | C_1_ | (0.000, 0.000, 0.000) |
|  | Nb_8_ | (0.667, 1.000, 0.945) | | C_2_ | (0.333, 0.667, 0.000) |
|  | Nb_9_ | (0.333, 0.333, 0.945) | | C_3_ | (0.667, 0.333, 0.000) |
|  | Nb_10_ | (0.000, 0.333, 0.445) | | C_4_ | (0.000, 0.000, 0.500) |
|  | Nb_11_ | (0.333, 1.000, 0.445) | | C_5_ | (0.333, 0.667, 0.500) |
|  | Nb_12_ | (0.667, 0.667, 0.445) | | C_6_ | (0.667, 0.333, 0.500) |
|  | Nb_13_ | (0.000, 1.000, 0.157) | | C_7_ | (0.000, 0.667, 0.109) |
|  | Nb_14_ | (0.333, 0.667, 0.157) | | C_8_ | (0.667, 1.000, 0.109) |
|  | Nb_15_ | (0.667, 0.333, 0.157) | | C_9_ | (0.333, 0.333, 0.109) |
|  | Nb_16_ | (0.000, 1.000, 0.657) | | C_10_ | (0.000, 0.333, 0.609) |
|  | Nb_17_ | (0.333, 0.667, 0.657) | | C_11_ | (0.333, 1.000, 0.609) |
|  | Nb_18_ | (0.667, 0.333, 0.657) | | C_12_ | (0.667, 0.667, 0.609) |
|  | Nb_19_ | (0.000, 1.000, 0.843) | | C_13_ | (0.000, 0.333, 0.891) |
|  | Nb_20_ | (0.333, 0.667, 0.843) | | C_14_ | (0.333, 1.000, 0.891) |
|  | Nb_21_ | (0.667, 0.333, 0.843) | | C_15_ | (0.667, 0.667, 0.891) |
|  | Nb_22_ | (0.000, 1.000, 0.343) | | C_16_ | (0.000, 0.667, 0.391) |
|  | Nb_23_ | (0.333, 0.667, 0.343) | | C_17_ | (0.667, 1.000, 0.391) |
|  | Nb_24_ | (0.667, 0.333, 0.343) | | C_18_ | (0.333, 0.333, 0.391) |

**Supplementary Table 2 |** Elastic constants *c_ij_* (GPa) of carbon-vacancy configuration VC8 and VC10.

|  | *c*_11_ | *c*_12_ | *c*_13_ | *c*_33_ | *c*_44_ | *c*_66_ |
| --- | --- | --- | --- | --- | --- | --- |
| VC8 | 372.9 | 109.9 | 135.7 | 329.3 | 158.1 | 131.5 |
| VC10 | 370.3 | 108.5 | 136.4 | 328.2 | 157.1 | 130.9 |

**Supplementary Table 3 |** Composition of the as-prepared Nb_4_AlC_3–_*_x_* (*x*  0.3). Averagely, Nb:Al = 4:1.05 (molar ratio).

|  | **Nb** | **Al** | **Nb:Al** |  | **Nb** | **Al** | **Nb:Al** |
| --- | --- | --- | --- | --- | --- | --- | --- |
| 1# | 48.52 | 12.60 | 4:1.04 | 16# | 48.9 | 12.70 | 4:1.04 |
| 2# | 48.89 | 12.92 | 4:1.06 | 17# | 48.92 | 12.89 | 4:1.05 |
| 3# | 48.81 | 12.86 | 4:1.05 | 18# | 49.14 | 13.05 | 4:1.06 |
| 4# | 48.10 | 12.82 | 4:1.07 | 19# | 48.82 | 12.90 | 4:1.06 |
| 5# | 48.56 | 12.76 | 4:1.05 | 20# | 49.34 | 12.93 | 4:1.05 |
| 6# | 48.39 | 12.86 | 4:1.06 | 21# | 48.5 | 12.77 | 4:1.05 |
| 7# | 48.61 | 12.67 | 4:1.04 | 22# | 48.84 | 12.77 | 4:1.05 |
| 8# | 48.53 | 12.88 | 4:1.06 | 23# | 48.75 | 12.55 | 4:1.03 |
| 9# | 48.76 | 12.73 | 4:1.04 | 24# | 48.96 | 12.88 | 4:1.05 |
| 10# | 48.71 | 12.82 | 4:1.05 | 25# | 48.96 | 12.72 | 4:1.04 |
| 11# | 48.62 | 12.74 | 4:1.05 | 26# | 48.97 | 12.89 | 4:1.05 |
| 12# | 47.89 | 12.79 | 4:1.07 | 27# | 48.86 | 12.92 | 4:1.06 |
| 13# | 48.6 | 12.85 | 4:1.06 | 28# | 48.99 | 12.82 | 4:1.05 |
| 14# | 48.89 | 12.96 | 4:1.06 | 29# | 48.98 | 12.92 | 4:1.05 |
| 15# | 48.55 | 12.97 | 4:1.07 | 30# | 48.55 | 12.99 | 4:1.07 |

**Supplementary Table 4** **|** Calculated X-ray diffraction data of o-Nb_4_AlC_3_ and Nb_4_AlC_3–_*_x_*. The superlattice peaks are highlighted in red.

| (*hkl*) | 2 () | I/Imax (%) | (*hkl*) | 2 () | I/Imax (%) | (*hkl*) | 2 () | I/Imax (%) | (*hkl*) | 2 () | I/Imax (%) |
| --- | --- | --- | --- | --- | --- | --- | --- | --- | --- | --- | --- |
| Nb_4_AlC_3–_*_x_* | | | o-Nb_4_AlC_3_ | | | Nb_4_AlC_3–_*_x_* | | | o-Nb_4_AlC_3_ | | |
| 002 | 7.31 | 60.63 | 002 | 7.32 | 51.95 |  | | | 204 | 41.21 | 0.03 |
| 002 | 7.33 | 30.31 | 002 | 7.33 | 25.97 |  |  |  | 204 | 41.32 | 0.01 |
| 004 | 14.66 | 12.58 | 004 | 14.66 | 14.12 |  |  |  | 1010 | 42.04 | 0.01 |
| 004 | 14.69 | 6.29 | 004 | 14.70 | 7.06 |  |  |  | 1010 | 42.14 | 0.00 |
|  | | | 100 | 18.88 | 0.09 | 107 | 42.35 | 13.91 | 117 | 42.36 | 14.65 |
|  |  |  | 100 | 18.93 | 0.04 | 107 | 42.46 | 6.95 | 117 | 42.47 | 7.33 |
|  |  |  | 102 | 20.27 | 0.05 |  | | | 206 | 44.64 | 0.07 |
|  |  |  | 102 | 20.32 | 0.02 |  |  |  | 206 | 44.75 | 0.04 |
| 006 | 22.06 | 4.46 | 006 | 22.07 | 4.56 | 108 | 44.88 | 4.76 | 118 | 44.90 | 4.46 |
| 006 | 22.12 | 2.23 | 006 | 22.12 | 2.28 | 0012 | 44.99 | 1.35 | 0012 | 45.01 | 1.21 |
|  | | | 104 | 23.99 | 0.15 | 108 | 45.00 | 2.38 | 118 | 45.01 | 2.23 |
|  |  |  | 104 | 24.05 | 0.07 | 0012 | 45.11 | 0.67 | 0012 | 45.13 | 0.61 |
|  |  |  | 106 | 29.20 | 0.00 | 109 | 47.62 | 2.25 | 119 | 47.64 | 2.06 |
|  |  |  | 106 | 29.27 | 0.00 | 109 | 47.75 | 1.12 | 119 | 47.76 | 1.03 |
| 008 | 29.56 | 13.74 | 008 | 29.57 | 14.90 |  | | | 208 | 49.11 | 0.09 |
| 008 | 29.63 | 6.87 | 008 | 29.64 | 7.45 |  |  |  | 1012 | 49.22 | 0.03 |
| 100 | 32.99 | 9.18 | 110 | 33.01 | 9.28 |  |  |  | 208 | 49.24 | 0.05 |
| 100 | 33.08 | 4.59 | 110 | 33.09 | 4.64 |  |  |  | 1012 | 49.35 | 0.02 |
| 101 | 33.21 | 12.55 | 111 | 33.22 | 12.88 | 1010 | 50.55 | 2.69 | 1110 | 50.57 | 3.07 |
| 101 | 33.29 | 6.27 | 111 | 33.30 | 6.44 | 1010 | 50.69 | 1.35 | 1110 | 50.70 | 1.54 |
| 102 | 33.84 | 41.97 | 112 | 33.85 | 46.41 |  | | | 210 | 51.43 | 0.02 |
| 102 | 33.93 | 20.98 | 112 | 33.94 | 23.21 |  |  |  | 210 | 51.57 | 0.01 |
| 103 | 34.87 | 23.41 | 113 | 34.88 | 26.19 |  |  |  | 211 | 51.58 | 0.03 |
| 103 | 34.96 | 11.70 | 113 | 34.97 | 13.09 |  |  |  | 211 | 51.72 | 0.01 |
|  | | | 108 | 35.32 | 0.57 |  |  |  | 209 | 51.82 | 0.00 |
|  |  |  | 108 | 35.41 | 0.29 |  |  |  | 212 | 52.03 | 0.05 |
| 104 | 36.27 | 43.16 | 114 | 36.29 | 47.24 |  |  |  | 212 | 52.17 | 0.03 |
| 104 | 36.37 | 21.58 | 114 | 36.38 | 23.62 |  |  |  | 213 | 52.76 | 0.61 |
| 0010 | 37.19 | 39.20 | 0010 | 37.20 | 42.17 |  |  |  | 213 | 52.90 | 0.30 |
| 0010 | 37.28 | 19.60 | 0010 | 37.30 | 21.08 | 0014 | 53.03 | 0.09 | 0014 | 53.04 | 0.06 |
| 105 | 38.01 | 60.53 | 115 | 38.02 | 62.61 | 0014 | 53.17 | 0.04 | 0014 | 53.19 | 0.03 |
| 105 | 38.11 | 30.27 | 115 | 38.12 | 31.30 | 1011 | 53.65 | 8.88 | 1111 | 53.67 | 9.01 |
|  | | | 200 | 38.30 | 0.01 |  |  |  | 214 | 53.78 | 0.01 |
|  |  |  | 200 | 38.39 | 0.00 | 1011 | 53.80 | 4.44 | 1111 | 53.82 | 4.50 |
|  |  |  | 202 | 39.04 | 0.70 |  | | | 214 | 53.92 | 0.00 |
|  |  |  | 202 | 39.14 | 0.35 |  |  |  | 2010 | 54.45 | 0.00 |
|  |  |  | 202 | 39.96 | 0.00 |  |  |  | 2010 | 54.60 | 0.00 |
| 106 | 40.05 | 100.00 | 116 | 40.06 | 100.00 |  |  |  | 215 | 55.07 | 0.02 |
| 106 | 40.15 | 50.00 | 116 | 40.16 | 50.00 |  |  |  | 215 | 55.22 | 0.01 |

**Supplementary Table 5 |** Theoretical Raman shifts of carbon-vacancy configuration VC10. The Raman shifts in red are believed to correspond to the extra Raman peaks marked by arrows in Figure 5c in the article. The experimental Raman shifts of those extra weak Raman peaks are provided in the parentheses.

| **Label** | **Raman Shift**  **(cm^–1^)** | **Symmetry** | **Label** | **Raman Shift**  **(cm^–1^)** | **Symmetry** |
| --- | --- | --- | --- | --- | --- |
| *ω*_1_ | 53 | *E*_2_ | *ω*_29_ | 263 | *E*_1_ |
| *ω*_2_ | 73 | *E*_2_ | *ω*_30_ | 263 | *E*_2_ |
| *ω*_3_ | 103 | *E*_1_ | *ω*_31_ | 267 | *E*_1_ |
| *ω*_4_ | 150 | *E*_1_ | *ω*_32_ | 267 | *E*_2_ |
| *ω*_5_ | 153 | *A*_1_ | *ω*_33_ | 276 | *A*_1_ |
| *ω*_6_ | 154 (154) | *A*_1_ | *ω*_34_ | 281 | *A*_1_ |
| *ω*_7_ | 169 | *E*_2_ | *ω*_35_ | 335 | *E*_1_ |
| *ω*_8_ | 170 (178) | *E*_2_ | *ω*_36_ | 335 | *E*_2_ |
| *ω*_9_ | 185 (181) | *E*_1_ | *ω*_37_ | 568 | *E*_2_ |
| *ω*_10_ | 187 (186) | *E*_1_ | *ω*_38_ | 569 | *E*_1_ |
| *ω*_11_ | 188 (186) | *E*_2_ | *ω*_39_ | 571 | *E*_1_ |
| *ω*_12_ | 190 | *E*_1_ | *ω*_40_ | 572 | *E*_2_ |
| *ω*_13_ | 191 | *E*_2_ | *ω*_41_ | 582 | *E*_1_ |
| *ω*_14_ | 193 | *E*_2_ | *ω*_42_ | 582 | *E*_2_ |
| *ω*_15_ | 196 | *E*_1_ | *ω*_43_ | 586 | *E*_1_ |
| *ω*_16_ | 204 | *E*_2_ | *ω*_44_ | 587 | *E*_2_ |
| *ω*_17_ | 204 | *E*_1_ | *ω*_45_ | 601 | *A*_1_ |
| *ω*_18_ | 206 | *A*_1_ | *ω*_46_ | 624 | *E*_2_ |
| *ω*_19_ | 223 | *E*_1_ | *ω*_47_ | 624 | *E*_1_ |
| *ω*_20_ | 224 | *E*_2_ | *ω*_48_ | 629 | *A*_1_ |
| *ω*_21_ | 227 | *E*_1_ | *ω*_49_ | 635 | *E*_1_ |
| *ω*_22_ | 227 | *E*_2_ | *ω*_50_ | 636 | *E*_2_ |
| *ω*_23_ | 229 | *E*_2_ | *ω*_51_ | 663 (660) | *E*_1_ |
| *ω*_24_ | 229 | *E*_1_ | *ω*_52_ | 663 (664) | *E*_2_ |
| *ω*_25_ | 230 | *A*_1_ | *ω*_53_ | 676 | *E*_1_ |
| *ω*_26_ | 236 | *E*_2_ | *ω*_54_ | 677 | *E*_2_ |
| *ω*_27_ | 236 | *E*_1_ | *ω*_55_ | 679 | *A*_1_ |
| *ω*_28_ | 252 | *A*_1_ |  |  |  |

**Supplementary Table 6 |** Crystal structure information of carbon-vacancy configuration VC10 optimized by first-principles.

| **Formula** | | Nb_12_Al_3_C_8_ |
| --- | --- | --- |
| **Space group** | | *P6*_3_22 (182) |
| **Lattice parameters (Å)** | | *a* = 5.486, *c* = 24.049 |
| **Atom positions** | Nb_1_ (4*e*) | (0.000, 0.000, 0.089) |
|  | Nb_2_ (12*i*) | (0.333, 0.017, 0.306) |
|  | Nb_3_ (4*f*) | (0.333, 0.667, 0.408) |
|  | Nb_4_ (4*f*) | (0.333, 0.667, 0.911) |
|  | Al (6*g*) | (0.338, 0.000, 0.500) |
|  | C_1_ (2*b*) | (0.000, 0.000, 0.250) |
|  | C_2_ (12*i*) | (0.333, 0.331, 0.359) |
|  | C_3_ (2*d*) | (0.333, 0.667, 0.750) |
